# THE ROLE OF EDUCATION AND LITERACY SKILLS ON MIDDLE-AGED AND OLDER VOLUNTEERS BY RACE AND ETHNICITY IN THE US

**DOI:** 10.1093/geroni/igac059.1430

**Published:** 2022-12-20

**Authors:** Donnette Narine, Takashi Yamashita, Wonmai Punksungka, Abigail Helsinger, Jenna Kramer, Rita Karam, Phyllis Cummins

**Affiliations:** University of Maryland, Baltimore, Baltimore, Maryland, United States; University of Maryland, Baltimore County, Baltimore, Maryland, United States; University of Maryland - Baltimore County, Baltimore, Maryland, United States; Miami University, Oxford, Ohio, United States; RAND Corporation, Arlington, Virginia, United States; RAND Corporation, Santa Monica, California, United States; Miami University, Oxford, Ohio, United States

## Abstract

Volunteer participation is a form of civic engagement that benefits both the individual and society over the life course. Although education, basic skills (e.g., literacy), and race/ethnicity are individually associated with volunteering, detailed interrelations are yet to be explored. Guided by the integrated theory of volunteer work and the notion of productive aging, the goal of this study was to examine the roles of education and adult literacy in the context of volunteering in later life across racial and ethnic groups (Whites, Blacks, Hispanics) in the U.S. Using the nationally representative sample of middle-aged and older adults (age 45+; n = 3,770) from the 2012/2014/2017 Program for International Assessment of Adult Competencies (PIAAC), structural equation modeling was constructed to evaluate mediation relationships among education, literacy, and volunteering by racial and ethnic groups. Results show no statistically significant mediation (a.k.a., indirect) effect of education on volunteering through literacy, nor was there statistically significant difference in the mediation effect across racial and ethnic groups. However, there were statistically significant differences in the direct effect of education on volunteering between Black adults and White adults [b(Black) = 0.44 versus b(White = 0.24), p < 0.05], as well as Black adults and Hispanic adults [b(Black) = 0.44 versus b(Hispanic) = 0.08, p < 0.05]. These findings indicate that higher education was more strongly associated with volunteering among older Black adults, compared to White and Hispanic counterparts. Suggested policy implications include support for the promotion of volunteer participation through culturally and socioeconomically sensitive approaches.

